# Prescriptions and Dispensing

**Published:** 1908-12-12

**Authors:** 


### Therapeutics.

### PRESCRIPTIONS AND DISPENSING.

### LOZENGES, PASTILLES, AND PASTES.

As we have already pointed out, our last few articles deal with questions which are mainly of interest to the medical man, in that he should know about the points discussed, although he is not so likely to make practical use of them himself, as he is in the case of the mixtures, pills, powders, and ointments discussed in our earlier articles.

It is sometimes required to administer certain medicaments in such a way that their action shall be exerted locally on the throat. This can, of course, be done in some cases by making them into a gargle, but another method, formerly much in vogue, and now regaining a popularity that has been in abeyance to some extent, is to employ a lozenge (trochiscus) or similar combination that can be slowly dissolved in the mouth.

The production of ordinary lozenges is not a dispensing operation, and will not be described here. There are, however, two other methods of preparing what is equivalent to a lozenge. The first is the production of compressed tablets as described above; the second is to prepare a sort of jujube or pastille in which the required medicament is combined with a slowly soluble basis. This method is not difficult, and it can be employed even for drugs that are not well suited for making into tablets.

The basis of pastilles is a stiff mass of gelatin with glycerin, which is commonly known as glyco-gelatine. The following is the formula from the Pharmacopœia of the Throat Hospital:—

| | | |
|---|---|---|
| ℞ | Gelatin ... ... ... ... ... | 1 oz. |
| | Glycerin ... ... ... ... ... | 2½ oz. |
| | Solution of Carmine, ammoniated ... | a sufficiency |
| | Orange flower water ... ... ... | 2½ fl. oz. |

The gelatin should be soaked in the orange flower water for two hours and then dissolved on a water-bath. The glycerin is then added and mixed well by stirring; the colouring solution is put in last, when the rest of the mixture has partly cooled.

Other flavouring agents may, of course, be used.

When required, the proper quantity of this basis is weighed out and melted on a water-bath; the medicament is then incorporated with it. If the drug is a soluble substance, cocaine hydrochloride for example, it can be dissolved in the basis and evenly distributed by stirring. The degree of heat used must be carefully watched so as not to destroy the activity of any ingredient put in. If it is not soluble it can be triturated to a smooth mixture with a little glycerin and water and then stirred in.

The mass is then poured out into a suitable tray, which may usually be extemporised from the lid of a tin, if necessary, and allowed to set. When cold it is taken out and cut with scissors into the correct number of equal portions.

Closely allied to pastilles are confections or electuaries, and mention may conveniently be made of them here. No gelatin is employed in making them; consequently the mixture does not set to a solid, but remains in the condition of a paste or jam, which is supplied to the patient in bulk, and the division into doses is made by the latter taking out the quantity ordered by means of a teaspoon or other rough measure. Glycerin may or may not be used. Confection of senna contains none, for example, whilst it is present in confection of sulphur.

Of the officinal representatives of this class of preparations Confectio Sennæ and Confectio Rosæ Gallicæ are seldom made except on the manufacturing scale. The latter has no medicinal properties, but is employed as a vehicle or excipient.

Confectio Piperis and Confectio Sulphuris, the two other pharmacopœial confections, are readily made by oneself. The absence of any special difficulty in their production is sufficiently indicated by the laconic nature of the directions, which consist of the one word "mix."

Confections of various other kinds may be devised and prescribed or dispensed. There is a tendency to avoid confections because of their approach to the remedies used by country people and other unqualified persons. This is a pity, however, for they are comparatively pleasant forms of medicine for patients to take; and a good deal can be learned by us from countrywomen and others if we will not at once turn a blind eye towards them because they are unqualified.

It is, of course, important that only fine powders should be employed, and these should be well mixed together before adding the liquid ingredients, the whole being well triturated together.

Professor Unna, of Vienna, has introduced into dermatological practice, particularly for the treatment of chronic ulcers of the legs, various pastes and jellies which are very similar to the above from the point of view of production. They differ mainly in being for external and not for internal application.

The following prescriptions are representative of the two classes:—

Iodine and Starch Paste.

| | | |
|---|---|---|
| ℞ | Pulveris Amyli ... ... ... ... | ʒj. |
| | Glycerini ... ... ... ... ... | ℥ij. |
| | Aquæ destillatæ ... ... ... ... | ℥vj. |
| | Liquoris Iodi (B.P. 1885) ... ... ... | ʒj. |

Triturate the starch with the glycerin and the distilled water; boil the mixture, and then allow to cool; when nearly cold, add the iodine solution and mix well.

Zinc Jelly.

| | | |
|---|---|---|
| ℞ | Gelatini ... ... ... ... ... | ʒj. |
| | Aquæ ... ... ... ... ... | ℥iiss. |
| | Zinci Oxidi ... ... ... ... ... | ʒj. |
| | Glycerini ... ... ... ... ... | ℥iiss. |

Soak the gelatin in the water for two hours; mix the zinc oxide well with the glycerine, and add these to the gelatin; heat on a water-bath till the gelatin has dissolved; stir well, and allow to cool.

Although properly a jelly, this preparation is generally known as Unna's paste. It is warmed till it is fluid before application, and put on with a brush over successive layers of bandage, setting into a firm and supporting case when cold.

*(To be continued.)*

